# Bibliometric analysis of acupuncture and moxibustion treatment for mild cognitive impairment

**DOI:** 10.3389/fnins.2023.1209262

**Published:** 2023-06-15

**Authors:** Wei Yang, Xingfang Liu, Xinyue Zhang, Cong Li, Zhenghong Li, Yiming Li, Mingquan Li

**Affiliations:** ^1^The Affiliated Hospital of Changchun University of Chinese Medicine, Changchun, China; ^2^Research Department, Swiss University of Traditional Chinese Medicine, Bad Zurzach, Switzerland; ^3^College of Integrated Chinese and Western Medicine, Changchun University of Chinese Medicine, Changchun, China; ^4^College of Traditional Chinese Medicine, Changchun University of Chinese Medicine, Changchun, China

**Keywords:** acupuncture and moxibustion, mild cognitive impairment, CiteSpace, VOSviewer, knowledge map, visual analysis

## Abstract

**Objective:**

This study aims to analyze the current research status of acupuncture in the treatment of mild cognitive impairment (MCI) using bibliometric methods, explore current research hotspots, and predict future research trends.

**Methods:**

Literature on acupuncture for MCI in China National Knowledge Infrastructure (CNKI) and Web of Science (WOS) databases were searched from their inception to December 31, 2022. Articles were then filtered using inclusion and exclusion criteria and imported into VOSviewer 1.6.11 and CiteSpace 6.1.6msi software for descriptive analysis of publication numbers, network analysis of author/institution collaborations, and cluster analysis of keywords, as well as analysis of keyword emergence and linear relationships with time.

**Results:**

The Chinese and English databases included 243 and 565 relevant articles, respectively. The overall volume of Chinese and English literature was stable, with the annual volume generally increasing. In terms of countries, institutions, and authors, China had the highest number of English-language publications; however, the number of joint publications among institutions/authors was low. Research institutions were independent and dispersed, with no collaborative teams formed around a single institution/author. The hotspots in Chinese literature were “needling, treatment, electric acupuncture, nimodipine, cognitive training” and other clinical research directions. The hotspots in English literature were “acupuncture, electro-acupuncture, Alzheimer’s disease, dementia, cognitive impairment, memory, vascular dementia, mild cognitive impairment, stroke, hippocampus, injury,” and other mechanisms of action.

**Conclusion:**

The popularity of acupuncture for MCI is increasing year by year. Acupuncture for MCI, along with cognitive training, can help improve cognitive function. “Inflammation” is the frontier of acupuncture for MCI research. In the future, strengthening effective communication and cooperation among institutions, especially international cooperation, is essential for conducting high-quality research on acupuncture for MCI. This will help obtain high-level evidence and improve the output and translation of research results.

## 1. Introduction

Mild cognitive impairment (MCI) is a transitional state between normal aging and dementia, characterized by cognitive decline and memory loss in some patients (Tangalos and Petersen, 2018). The prevalence of MCI in adults over 60 years of age is approximately 6.7 to 25.2% (Jongsiriyanyong and Limpawattana, 2018) and increases with age. Acupuncture stimulation of corresponding acupoints can improve blood circulation (Yuan et al., 2022) and help balance Yin and Yang while dredging meridians, thereby intervening in patients’ cognitive function and promoting cognitive recovery (Kuang et al., 2021). Recently, traditional Chinese medicine (TCM) acupuncture has made significant progress in treating MCI (Liu et al., 2023). With increasing research in this field, some reviews have discussed acupuncture treatment for MCI, but no scholars have conducted a systematic analysis of the profile, hotspots, and frontier of acupuncture treatment using visual analysis.

Bibliometrics refers to the interdisciplinary science that employs quantitative mathematical and statistical methods (Chen et al., 2021). It is a comprehensive knowledge system that integrates mathematics, statistics, and philology while emphasizing quantification. In particular, the application of information visualization technology and methods can intuitively display the research development process, current research status, research hotspots, and development trends. CiteSpace and VOSviewer are the most widely used software tools for literature information visualization.

VOSviewer (van Eck and Waltman, 2010) and CiteSpace (Li et al., 2022) are visual analysis software developed by Professor Van Eck of Leiden University in the Netherlands and Professor Chen Chaomei of Drexel University in the United States, respectively. VOSviewer primarily deconstructs the relationships of elements to be analyzed by distance, while CiteSpace focuses on graphics and connections, showing the strength of the relationships among the analyzed elements. The main features of both are their rich graphical presentations and clear displays, making the results of bibliometric analyses easy to interpret.

This study, based on China National Knowledge Infrastructure (CNKI) and Web of Science (WOS) databases, applies bibliometric methods and uses CiteSpace and VOSviewer software to visually analyze literature information. This approach is more intuitive and comprehensive than traditional reviews and clinical research, as it identifies the research hotspots of acupuncture and moxibustion treatment for MCI and provides insights for future research.

## 2. Data and methods

### 2.1. Literature sources and data retrieval strategy

For Chinese literature in the CNKI database, the search formula is: SU = (‘'针'+'针灸'+'灸'+'电针'+'毫针'+'火针'+'腕踝针'+'眼针'+'揿针'+'蜂针'+'舌针'+'腹针'+'耳针'+'头针'+'体针'+'针法'+'艾灸') AND SU = ('轻度认知障碍'+'轻度认知损伤'+'轻度认 知损害 + ‘MCI’ + ‘SCD’). For English literature in the WOS database, the search formula is: TS = (acupuncture OR pharmacopuncture) AND TS = (mild cognitive impairment OR Cognitive Dysfunctions* OR Cognitive Impairment* OR Cognitive Disorder* OR Mild Cognitive Impairment OR Cognitive Decline* OR Mental Deterioration*). The search period ranges from the inception of the databases to December 31, 2022.

### 2.2. Literature inclusion and exclusion criteria

Inclusion criteria for Chinese literature: (1) literature source: CNKI; (2) literature on acupuncture for MCI. Exclusion criteria: duplicate papers, conference papers, scientific and technological achievements, and newspaper literature.

Inclusion criteria for English literature: (1) literature source: WOS; (2) literature on acupuncture treatment for MCI. Exclusion criteria: duplicate papers, conference papers, scientific and technological achievements, and newspaper documents.

### 2.3. Literature screening method

Two researchers independently screened the literature by reading the titles and abstracts. They excluded literature that did not meet the criteria and performed a cross-check. For any divergent literature, the decision was made through discussion.

### 2.4. Visual analysis

#### 2.4.1. Literature data extraction

The screened Chinese literature is exported from CNKI in Refworks format, named as “download##.txt” for source data processing; the filtered English literature is exported in “plain text” file format and named as “download##.txt” for source data processing. The data to be analyzed include literature titles.

#### 2.4.2. Visual software

Chinese literature is primarily analyzed using CiteSpace-6.1.6msi, while English literature employs CiteSpace-6.1.6msi and VOSviewer 1.6.11 software.

#### 2.4.3. Literature cluster analysis

Variables such as authors, institutions, and keywords are extracted, followed by cluster analysis. The Chinese literature keyword cluster uses the LLR algorithm module in CiteSpace-6.1.6msi to draw the visual map of MCI keyword analysis for acupuncture treatment. In the keyword cluster analysis of English literature, the three visualization modules provided by VOSviewer—network visualization, overlay visualization, and density visualization—are selected for cluster analysis to generate the keyword cluster map.

##### 2.4.3.1. Network visualization

Circles and labels of an element represent its size, which depends on the node degree, connection strength, citations, etc. The element’s color represents its cluster, with different clusters in different colors. Through the view, one can examine each individual cluster, discover research hotspots through thematic co-occurrence, research communities through author collaboration, and similarities and differences between scholars on research topics through author coupling networks.

##### 2.4.3.2. Overlay visualization

Nodes are assigned different colors based on the score or color (red, green, blue) fields in the map file. By default, the average year of the keyword is used for color mapping.

##### 2.4.3.3. Density visualization

Each point on the map is filled with a color according to the density of the surrounding elements. Higher density areas are closer to red, while lower density areas are closer to blue. Density size depends on the number of elements in the surrounding region and the importance of these elements. Density view can be used to quickly observe important areas and the density of knowledge fields and studies.

CiteSpace provides two metrics, Modularity (Q value) and Weighted Mean Silhouette (S value), which serve as a basis for judging the mapping’s effectiveness. Generally, a Q value in the [0,1] range, Q > 0.3, indicates that the structure of the delineated associations is significant. An S value of 0.7 indicates that the clustering is efficient and convincing. If it is above 0.5, the clustering is usually considered reasonable.

#### 2.4.4. Investigator cooperative network visualization analysis

In the research section, Price’s Law (Wang et al., 2022) *N*=0.749 × $\sqrt{\text{max}}$ is used to determine the number of core author publications.

#### 2.4.5. Visualization of social network analysis graphs

In the visual social network analysis diagram, the size of each node represents the frequency of the analyzed variable; the edge represents the connection between variables; two variables appearing together in the same document will have an edge; the thickness of the line between nodes indicates the strength of the association; different colors indicate different clusters.

#### 2.4.6. Keyword timeline atlas analysis

The timeline view shows the keywords according to time, indicating the hot evolution and stage characteristics of keywords in the field (Qi et al., 2020), we can analyze the frequency and growth rate of keywords and their clusters from the perspective of time and explore the hot research issues to predict the development trend (Wei et al., 2021). Keywords of the same cluster were placed on the same horizontal line, with the time corresponding to the keywords positioned at the top of the view. In the time line map, the more keywords there are, the more important the clustering field is. The size of the circle in the figure represents the key word. The larger the circle, the higher the frequency number.

#### 2.4.7. Keyword emergence analysis

Keyword emergence is a rapid increase in the frequency of keywords within a certain period (Yan et al., 2020; Gao et al., 2021). The blue lines in the figure indicate the start and end time of the keywords, while the red lines indicate the time period from the keyword emergence to the end.Click “Burstness” in the control panel to detect emerging words, and click “Refresh” to calculate the number of upcoming words. If the number of non-emergent words or emergent words is too small, the value of Y [0,1] can be reduced until sufficient number of emergent words are obtained.

## 3. Results

**FIGURE 1:**
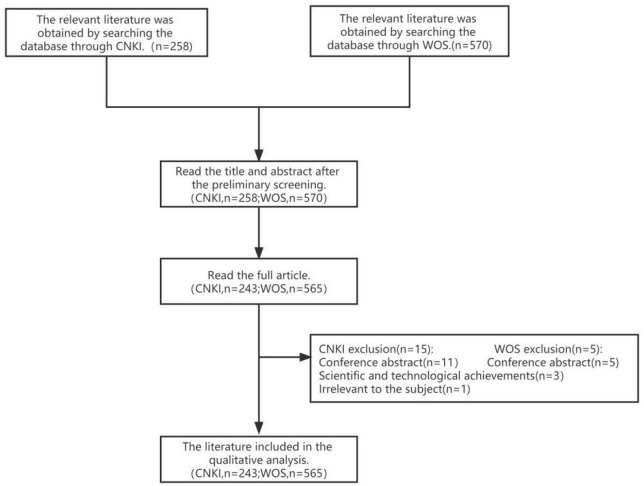
Flow chart of literature screening.

A total of 258 Chinese documents were retrieved from CNKI, and 243 of them were included after literature screening. Additionally, 570 English documents were retrieved from WOS, and 565 of them were selected for further analysis (Figure 1).

### 3.1. Visual analysis results of the Chinese literature

#### 3.1.1. Statistics on the number of articles published

**FIGURE 2:**
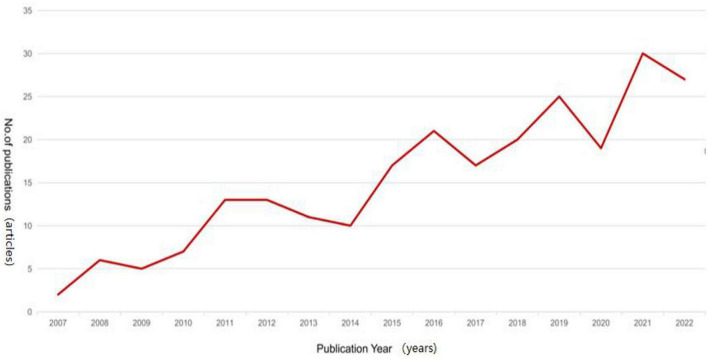
Trend chart of the annual publications of Chinese literature.

Among the 243 selected Chinese articles, the first one was “30 cases of mild cognitive impairment treated by the combination of the Yuanluotongjing acupuncture method and oral administration of An Lishen” published in the Chinese Journal of Chinese Medicine Science and Technology in 2007. According to Figure 2, there was an overall upward trend in the number of annual publications from 2007 to 2022. The lowest number of publications was in 2007, with two articles, while the highest number of publications was in 2021, with 30 articles (Figure 2).

In terms of publication types, academic journals accounted for the majority of articles (159 articles), followed by doctoral and master’s degree theses (84 articles). The top three journals in terms of number of publications were the Shanghai Acupuncture Journal (6.918%), Chinese Acupuncture (4.348%), and Clinical Journal of Chinese Medicine (4.348%). The top 10 journals in terms of number of publications are shown in Table 1. Approximately 24.28% of the articles were published in the above-metioned 10 journals, which mainly focused on traditional Chinese medicine, acupuncture and moxibustion, and traditional Chinese pharmacy, etc.

**TABLE 1:** Top 10 magazines in terms of publications.

Publication	Number of literature (articles)	Proportion (%)
Shanghai Acupuncture magazine	11	6.92
Chinese acupuncture	7	4.40
A Clinical Journal of Traditional Chinese Medicine	7	4.40
The Chinese Journal of Gerontology	6	3.77
Clinical Journal of Acupuncture	6	3.77
Journal of Anhui University of Traditional Chinese Medicine	5	3.15
New traditional Chinese medicine	5	3.15
The Chinese Journal of Traditional Chinese Medicine	4	2.52
Asia Pacific Traditional Medicine	4	2.52
Yunnan Journal of Traditional Chinese Medicine	4	2.52

#### 3.1.2. Research institution network analysis

**FIGURE 3:**
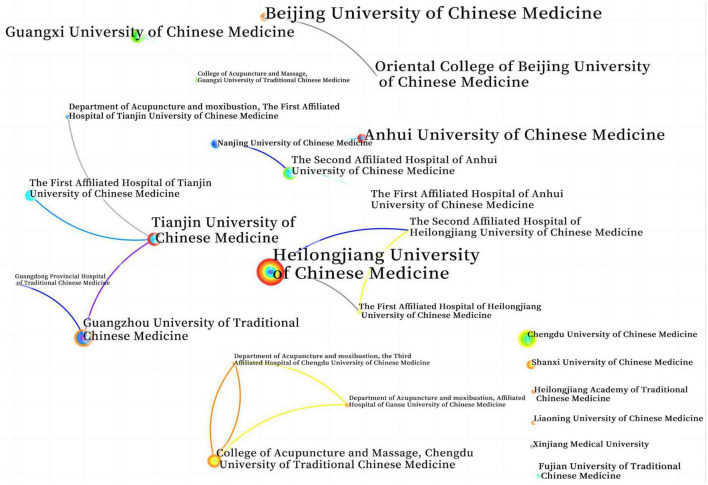
Network analysis atlas of the research institution (frequency ≥ 3 articles).

Network analysis of the research institutions showed that there were 204 nodes, 188 edges, and a density of 0.0091. The top five institutions with the largest number of articles are: Heilongjiang University of Chinese Medicine (25 articles), Guangzhou University of Chinese Medicine (17 articles), Tianjin University of Chinese Medicine (15 articles), Chengdu University of Chinese Medicine (13 articles), and Guangxi University of Chinese Medicine (11 articles). Among them, 25 institutions had three or more publications, accounting for 12% of the total number of institutions. The relationship of the published literature network of each research team is shown in Figure 3, indicating that various institutions have little cooperation in MCI research, mainly independent research, and most of them are traditional Chinese medicine universities.

#### 3.1.3. Author partnership network analysis

**FIGURE 4:**
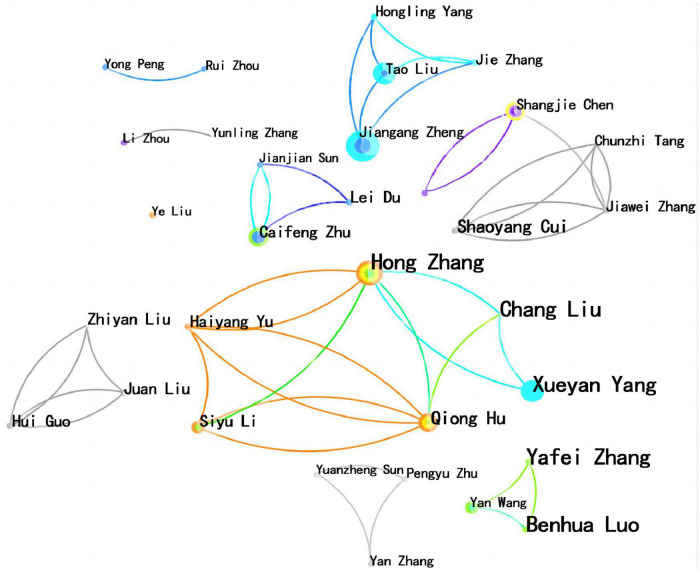
Network analysis atlas of the author (frequency ≥ 3 times).

Network analysis with authors as nodes yielded 339 nodes, 604 edges and a density of 0.0106. Among the included literature, 33 authors had more than 3 publications, accounting for 10% of the total number of authors. The top 5 authors in terms of publications were Zhang Hong, Hu Qiong, Zheng Jiangang, Zhu Caifeng, and Chen Shangjie, with Zhang Hong having the highest number of publications at 13. A network visualisation of these author collaborations (Figure 4) suggests a relatively fragmented group of authors, with the larger being a core group of authors centred on Zhang Hong.

#### 3.1.4. Keyword analysis

**FIGURE 5:**
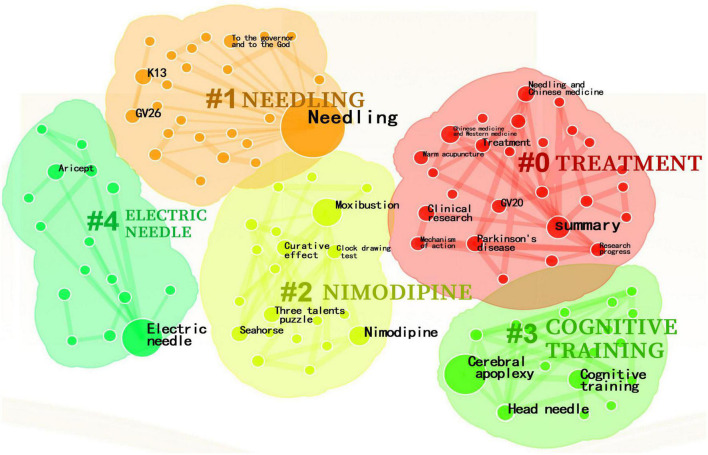
CNKI keyword cluster map (frequency ≥ 3 times).

**FIGURE 6:**
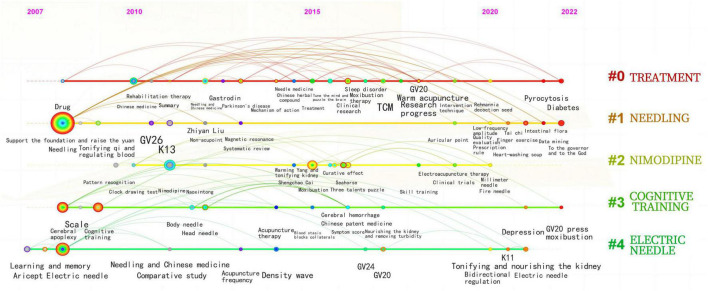
Chinese literature keyword time line chart.

Through cluster analysis of Chinese literature keywords, Q value = 0.8382, S value = 0.9673, a total of 5 class keywords were identified, mainly related to needling, treatment, electric acupuncture, nimodipine, cognitive training (Figure 5). Based on the cluster map, a timeline was constructed (Figure 6). The earliest keywords was “aricept”, which were studied for a long period of time, with the research focus mainly on “treatment”, “needling”, “cognitive training” and “electric acupuncture”.

#### 3.1.5. Keyword emergent analysis

**FIGURE 7:**
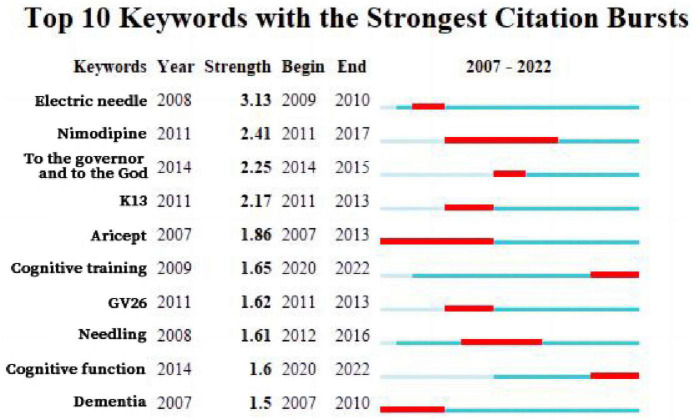
Present map of CNKI keywords.

Emergent analysis of keywords in CNKI Chinese literature generated 10 emergent words (Figure 7). Among them, the earliest emergence time was “aricept” and “dementia”, which appeared in 2007. The keywords “nimodipine” and “aricept” had a longer emergence time, while “electric needle” had the strongest intensity, with an intensity of 3.13. The keywords “Cognitive training” and “Cognitive function” are still being studied today.

### 3.2. Visual analysis of literature based on Web of Science

#### 3.2.1. Statistics on the number of articles published

**FIGURE 8:**
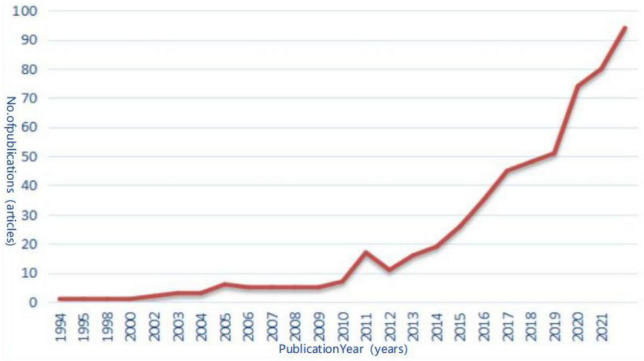
Trends in the annual number of articles published in English.

The first relevant paper was “TENSION-TYPE HEADACHE - PSYCHOSOMATIC CLINICAL-ASSESSMENT AND TREATMENT” published by BIONDI, M, an Italian, in the journal Psychotherapy and Psychosomatics in 1994. Over the past 30 years, the number of articles published on the topic of acupuncture and moxibustion for MCI has increased year by year. From 1994 to 2010, the number of articles on the topic of acupuncture for MCI began to increase each year, but the trend was slow. From 2012 to 2022, the number of articles increased significantly over the 10-year period, with a rapid increase in the number of articles on topics related to acupuncture for MCI starting in 2019 (Figure 8).

The type of literature was dominated by original research (66%), followed by review articles (30%). The top three journals in terms of number of publications were Medicine (38 articles, 6.73%), Evidence based complementary and alternative medicine (35 articles, 6.20%), and Trials (21 articles, 3.72%). The top 20 journals in terms of number of publications are shown in Table 2. Nearly half (43.01%) of the literature was published in these 20 journals in the areas of neuroscience, Chinese medicine, acupuncture and moxibustion, complementary alternative medicine, psychiatry, and cell biology.

**TABLE 2:** Top 20 journals in terms of number of articles published.

Publication	Number of literature (articles)	Proportion (%)
Medicine	38	6.73
Evidence Based Complementary and Alternative Medicine	35	6.20
Trials	21	3.72
Acupuncture in Medicine	18	3.19
Frontiers in Aging Neuroscienced	15	2.66
BMC Complementary and Alternative Medicine	12	2.12
Neural Regeneration Research	11	1.95
Cochrane Database of Systematic Reviews	10	1.77
Frontiers in Neurology	9	1.59
Journal of Alternative and Complementary Medicine	9	1.59
Neural Plasticity	8	1.42
PLOS One	8	1.42
World Journal of Acupuncture Moxibustion	8	1.42
BMJ Open	7	1.24
Journal of Acupuncture and Tuina Science	7	1.24
Explore the Journal of Science and Healing	6	1.06
Frontiers in Psychology	6	1.06
European Journal of Integrative Medicine	5	0.89
Frontiers in Neuroscience	5	0.89
Journal of Nervous and Mental	5	0.89

#### 3.2.2. Visual analysis of the network of issuing countries/regions and issuing institutions

**FIGURE 9:**
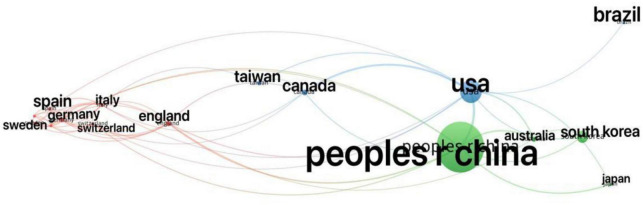
Analysis of country/regional cooperation in published literature.

**FIGURE 10:**
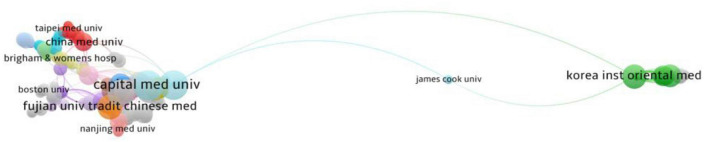
Social network analysis graph for academic institutions with published literature.

The top ten countries in terms of number of publications are shown in Table 3, with China being the country with the highest number of publications. An analysis of country/region cooperation in published literature is shown in Figure 9, and there is a need to strengthen cooperation between countries. The top 12 research institutions in terms of number of publications are shown in Table 4, with 77 of these academic institutions being from China. The Capital Medical University holds the top position with 47 publications. However, there are few international collaborations on MCI research in the mapping of institutional collaboration networks, with only one collaboration between Capital Medical University and the Toyo Institute in Korea (Figure 10).

**TABLE 3:** Top 10 countries in terms of number of articles published.

Country	Number of literature (articles)
Peoples R China	328
USA	118
South Korea	51
Canada	20
Australia	16
England	16
Germany	15
Taiwan	15
Italy	8
Italy	6

**TABLE 4:** Academic institutions with published literature.

Name of Institution	Number of literature (articles)
Capital Medical University	47
Beijing University of Chinese Medicine	45
Guangzhou University of Chinese Medicine	35
Fujian University of Traditional Chinese Medicine	29
Tianjin University of Traditional Chinese Medicine	26
Korea Institute of Oriental Medicine KIOM	25
University of Hong Kong	21
Kyung Hee University	19
Shanghai University of Traditional Chinese Medicine	19
Harvard University	18
China Academy of Chinese Medical Sciences	16
Zhejiang Chinese Medical University	16

#### 3.2.3. Researcher posting volume and network visualization analysis

**FIGURE 11:**
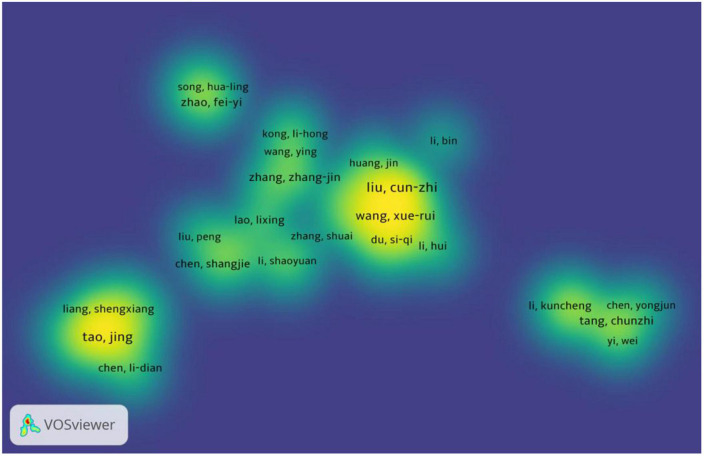
Analysis of the core author collaboration network.

A total of 2693 authors were involved in the 565 publications, with 12 authors having ≥ 10 publications, all from China. The authors with the highest number of publications are Liu, Cun Zhi and Chen, Lidian, with each author having published 20 articles. According to Price’s law N ≈ 3, the authors with 3 publications are the core authors in the field. The number of authors with three publications was 162. Using VOSviewer to plot the density view of the core author collaboration network (Figure 11), six author groups were formed, most of which consisted of 5-6 authors, but the fusion component of each author group was low, suggesting that there were fewer links between the teams.

#### 3.2.4. Visual analysis of keyword clustering

**FIGURE 12:**
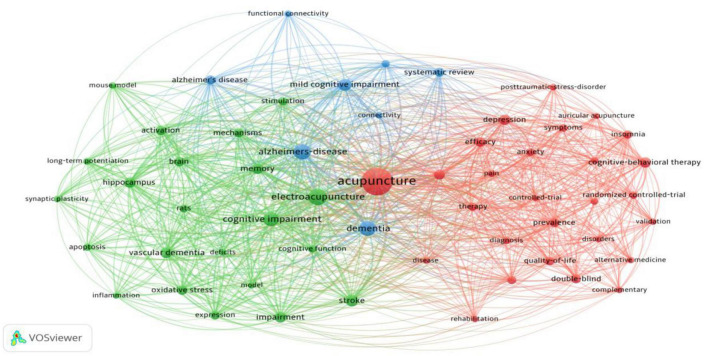
Keyword clustering map.

The keyword-based social network analysis is shown in Figure 12, with the main keyword clusters grouped into three broad categories:

The red area is cluster 1, with core cluster terms such as acupuncture, auricular acupuncture, cognitive behavioural therapy, randomised controlled, double-blind, efficacy, meta-analysis, anxiety, and depression, etc. The clustering themes are clinical treatments common to acupuncture for MCI, research methods, and MCI for concomitant diseases.

Cluster 2, the green area, has core terms such as electroacupuncture, cognitive impairment, memory, mechanism, stroke, vascular dementia, hippocampus, apoptosis, inflammation, oxidative stress, rat models, etc. The theme is research on mechanisms related to acupuncture for MCI in animal experiments.

Cluster 3, the yellow area, has core terms such as dementia, Alzheimer’s disease, mild cognitive impairment, systematic review, etc. The theme is MCI-related diseases and popular research trends.

**FIGURE 13:**
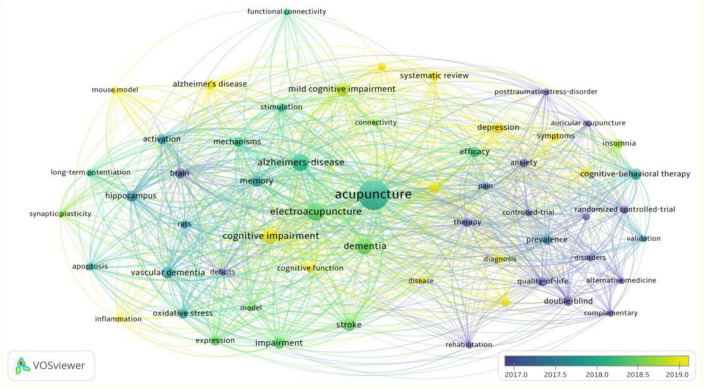
Social network timing diagram for keywords.

A temporal analysis of social networks based on keywords shows that acupuncture appears earlier and more frequently than MCI, and that research on acupuncture for Alzheimer’s disease or other neurological disorders predates research on acupuncture for MCI (Figure 13).

#### 3.2.5. Analysis of keyword emergence

**FIGURE 14:**
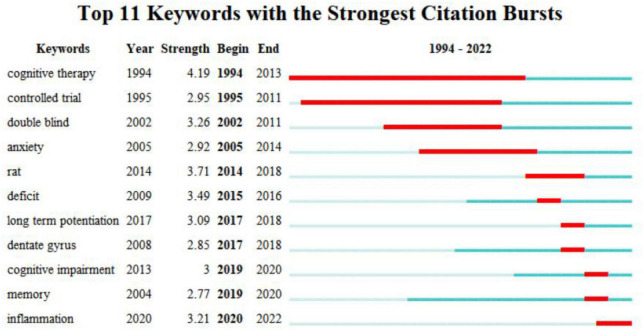
Keyword emergence map of English literature.

The keyword emergence analysis of the WOS English literature generated 11 emergent terms (Figure 14). “Cognitive therapy” was the first (1994), strongest (4.19), and longest lasting keyword (20 years) to emerge. This was followed by “controlled trials”, “double-blind” “anxiety” and “rat”, which emerged with a relatively long duration. “Long-term enhancement” and “dentate gyrus” began to appear in 2017 and lasted for 2 years. “Cognitive impairment” and “memory” are words that have emerged in recent years but not for long. “Inflammation” emerged in 2020 and continues today, and is considered to be at the forefront of research on acupuncture for MCI.

## 4. Discussion

### 4.1. Acupuncture for MCI research requires greater collaboration between institutions and researchers

Studies have shown that the conversion rate of MCI patients to dementia is between 8 and 15% (Jin et al., 2022). It is of great significance to prevent and treat AD during the MCI Stage. Acupuncture, as a traditional Chinese medicine treatment, has the advantages of being safe, convenient and effective, making it a potential future trend for MCI treatment.

The analysis of the number of articles published reflects the main trends in this research area (Cheng et al., 2006). The results of this study show a significant increase in Chinese literature since 2014 and a significant increase in the English literature in WOS since 2012, suggesting a gradual increase in research interest. Studies in the English literature were mainly conducted in China, emphasizing the promotion of the advantages of acupuncture for MCI worldwide.

However, analysis of institutional/author social networks showed that there were few joint studies within China or internationally, the researcher teams were relatively independent and mainly from various TCM universities, the types of studies were mostly single-centre small-sample clinical studies, and differences in the selection of representative samples, sample sizes, and interventions all contributed to the limitations of the studies to some extent. In recent years, as the status of acupuncture and moxibustion in international medicine has increased, scholars in more and more countries have focused on the effectiveness of acupuncture and moxibustion in the treatment of diseases (Su and Wang, 2021). Future research will require greater collaboration not only between research teams within the country, but also between international institutions. To do this, efforts can be made in two ways: The first is to give full play to the guiding role of the government and various academic groups to provide policy and project support for acupuncture and moxibustion treatment in MCI, to expand the scope of cooperation in different disciplines, specialties and geographical areas, and to encourage multi-centre, high-quality clinical research, so as to form a rich and stable cooperative network of researchers and research institutions in this field as soon as possible. The second is to build international or domestic academic exchange platforms, create opportunities for cooperation through increased exchanges, encourage more researchers to participate in research in this field, and continue to make deeper and more detailed and solid research results, so as to strengthen the core group of researchers in this field. Multi-centre, large sample and high quality clinical research is the future trend. Strengthening cooperation between different countries, institutions and scholars to achieve complementary advantages, information sharing and resource sharing will make the research content and findings more in-depth and scientific and help create more high quality evidence.

### 4.2. Differences in research hotspots in the English and Chinese literature on acupuncture and moxibustion for MCI

Keywords reflect the focus of literature, with high frequency keywords representing research hotspots and trends to some extent (Zhou et al., 2022). Visual analysis of keywords in Chinese literature showed a focus on needling, treatment, electric acupuncture, nimodipine and cognitive training, with a primary emphasis on clinical research on acupuncture for MCI. In contrast, analysis of keywords in the English literature showed apoptosis, inflammation, and oxidative stress as core keywords, indicating a focus on research results in the mechanism of action category. Future publications should focus on the publication of clinical research findings in international journals.

Analysis of the keyword timeline and emergence revealed that needling, treatment, electric acupuncture, cognitive training were popular areas of research, while nimodipine were relatively popular areas of research. Studies in the Chinese literature from 2007 to 2010 focus on needling and electroacupuncture for MCI, with studies after 2011 refining acupuncture and moxibustion for MCI and the use of medication in combination therapy. In 2020, studies began to focus on acupuncture and moxibustion with cognitive training therapy for cognitive impairment, which remains a hot research topic. Emergent analysis of Keywords in English literature suggests a shift in focus from clinical randomized controlled trials to transgenic mouse animal studies between 1994 and 2022. Researchers shifting their research focus to animal studies based on clinical studies to explore and confirm structural pathological changes in brain function, mechanistic exploration, and associated factors in the treatment of MCI with acupuncture through animal studies, but there is still a lack of evidence from high-quality clinical studies.

### 4.3. International hotspots and trends in the mechanism of action of acupuncture and moxibustion for MCI

Keyword analysis suggests that acupuncture and moxibustion mechanisms in the treatment of MCI is a current research hotspot (Zhang et al., 2013; Petersen, 2016). Analysis of the published literature on acupuncture and moxibustion mechanisms in treating MCI (Li et al., 2021; Yin et al., 2023)suggests that acupuncture and moxibustion is closely related to the neurotoxic mechanism of Aβ, the mechanism of oxygen free radical damage, the mechanism of cytokine-induced inflammatory response, and the mechanism of cerebral blood perfusion (Yang et al., 2021). Acupuncture can improve cognition by modulating neurotransmitter levels, neuroinflammatory response, and regulating the expression of related genes and brain proteins.

Some studies have shown that electroacupuncture at the “GV20” and “ST36” acupoints can inhibit the inflammatory response mediated by the NLRP3/Caspase-1 signaling pathway, reduce the serum levels of IL-1β, IL-6, IL-18, and TNF-α, down-regulate the protein expression levels of NLRP3, ASC, Caspase-1, GSDM-D, IL-1β, and IL-18 in hippocampal tissues, thereby inhibiting the scorching of hippocampal neuronal cells and effectively improving the learning and memory abilities of SAMP8 mice (Li, 2022). Electroacupuncture at the Baihui and Renyu acupoints was found to inhibit the hyperphosphorylation of Tau protein, as evidenced by the observed downregulation of p-38MAPK and p-tau Thrl81 protein expressions in the hippocampus of rats (Zhang et al., 2015). The “Sancai Puzzle” moxibustion method can also improve the cognitive function and reduce the levels of serum Aβ1-42, Tau, and P-tau in a MCI patients, thus inhibiting the phosphorylation level and improving their learning and memory ability (Wang et al., 2021). Acupuncture and moxibustion treatment for MCI can have a calming effect on the brain by maintaining cognitive function and increasing the connection between the heart, kidneys, and brain. Cholinergic neurons are crucial for the hippocampal loop of learning and memory in the brain, and their damage leads to dementia. Acupuncture and moxibustion can improve cognitive impairment by improving the abnormal state of the cholinesterase system and regulating synaptic plasticity in the hippocampus (Yan et al., 2015; Song et al., 2017). Henan Sun found that the plasma Ach levels in the observation group were significantly higher than those in the control group before and after treatment, and the MMSE and HDS scale scores were positively correlated with plasma Ach levels (Sun et al., 2018).

“Inflammation”, a keyword that emerged in 2020 and continues to be used today, is currently the main focus of research in acupuncture and moxibustion for MCI. The pathogenesis of MCI and AD is multifaceted, with the presence of neuroinflammation thought to be one of the driving factors that affect the progression of normal ageing to MCI and MCI to AD. Chronic inflammation of the central nervous system is considered a key factor in the progression of neurodegenerative diseases. Age-related neuroinflammation is characterized by increased levels of pro-inflammatory cytokines, such as interleukin (IL)-1β, as suggested by Norden and Godbout (2013). Acupuncture and moxibustion can improve learning and memory by modulating TNF-α, IL-1β, and IL-6 in the frontal cortex and reducing the damage to brain tissue caused by inflammatory factors. This inflammatory state may result from the activation of glial cells and astrocytes in the ageing brain (Feng et al., 2018) which coincides with an increase in systemic inflammation, that may trigger neuroinflammation, further impairing cognitive abilities (Anderson, 2022).

### 4.4. Study limitations

The study had limitations as it only searched two databases, CNKI and WOS, and may not have included all relevant studies in this field of acupuncture for MCI. Thus, the results of the analysis have some limitations, but they can still reflect the current research status and future trends in this field to a certain extent. It is hoped that subsequent analysis will cover a wider range of literature and provide more Comprehensive references and evidence for future studies on the hotspots and trends of acupuncture and moxibustion for MCI.

## 5. Conclusion

In summary, this article presents a visualization and analysis of relevant literature in the field of acupuncture and moxibustion for MCI in the past 30 years using CiteSpace and VOSviewer software. The study suggests that acupuncture and moxibustion for mild cognitive impairment is becoming increasingly popular each year, that acupuncture and moxibustion in conjunction with cognitive training may help improve cognitive function, and that “inflammation” is at the forefront of acupuncture and moxibustion research for MCI. Follow-up studies are needed to strengthen effective communication and cooperation among institutions, especially international cooperation, to conduct more high-quality studies on acupuncture and moxibustion for MCI and improve the output and translation of scientific results.

## Data availability statement

The original contributions presented in this study are included in the article/supplementary material, further inquiries can be directed to the corresponding authors.

## Author contributions

WY and XL contributed to conception and design of the study. ZL organized the database. CL performed the statistical analysis. XZ wrote the first draft of the manuscript. ML and YL wrote sections of the manuscript. All authors contributed to the article and approved the submitted version.
